# Effects of dietary energy and protein levels on nutrient intake, digestibility, and body weight change in Hararghe highland and Afar sheep breeds of Ethiopia

**DOI:** 10.5455/javar.2021.h501

**Published:** 2021-05-19

**Authors:** Gadissa Sileshi, Eshetu Mitiku, Urge Mengistu, Tolera Adugna, Feyissa Fekede

**Affiliations:** 1 School of Animal and Range Sciences, College of Agriculture and Environmental Sciences, Haramaya University, Dire Dawa, Ethiopia; 2 School of Animal and Range Science, College of Agriculture, Hawassa University, Awassa, Ethiopia; 3 Ethiopian Institute of Agricultural Research, Addis Ababa, Ethiopia

**Keywords:** Average daily gain, digestibility, dry matter intake, feed conversion efficiency, nutrients intake

## Abstract

**Objectives:**

The experiment was conducted to determine the effect of dietary energy and protein level growth performances of selected indigenous Ethiopian sheep breeds.

**Materials and Methods:**

Fifty intact ram lambs, 25 from each breed with 12 months of age and a mean initial body weight (IBW) of 19.31 ± 1.7 kg, were employed for this experiment. Animals were distributed randomly into five dietary treatments, i.e., minimum Energy and Protein (mEmP), medium energy and protein (MEMP), medium Energy and high Protein (MEHP), high energy and medium protein (HEMP), and high Energy and high Protein (HEHP) diets in randomized complete block design with 2 * 5 factorial arrangements. The minimum, medium, and high energy diets were 2.388, 2.866, and 3.344 Mcal/kg dry matter (DM) with the corresponding 10%, 16%, and 20% crude protein (CP) diets, respectively. The diets were formulated in a total mixed ration from wheat bran (WB), maize grain, peanut cake, and pasture hay feed ingredients. Diet offer was at the rate of 3% of lambs’ live weight and revised biweekly as per the attained body weight changes. Digestibility trial was conducted for 7 days of actual fecal data collection, followed by 90 days of feeding trial.

**Results:**

The animals fed on the MEHP diet had a maximum DM and nutrient intakes (CP and organic matter) and the best final body weight (FBW), total gain, gain rate, average daily gains, and feed conversion efficiency (31.3, 12.9 kg, 41.2%, 143.3 gm, and 23.13, respectively), followed by HEMP, HEHP, MEMP, and mEmP diets. Digestibility of DM and nutrients linearly followed similar trends (*p* < 0.01). Hararghe sheep was heavier (*p* < 0.01) by 4.3 and 3.1 kg in its FBW and total gain and more efficient in nutrients utilization (22.57 *vs.* 18.18) as compared to Afar sheep (AS).

**Conclusion:**

It is concluded that MEHP and MEMP are superior and optimum diets for sheep breeds, and Hararghe sheep is carried out better than AS in most growth performance parameters.

## Introduction

Sheep (*Ovis aries*) are commonly reared in mixed farming systems and found in Ethiopia’s agro-ecological zones [1]. However, their growth performance and productivity are low due to inadequate feedstuffs and nutrition in both quantity and quality [2,3]. In the country, farm animals are fed on natural pasture and crop residues. However, these feedstuffs have poor nutritive values and do not meet animals’ nutrient requirements [4]. Proper growth performance in ram lambs depends mainly on a nutritional level. Thus, protein and energy sources’ inclusion in the diets would help the rumen environment and microbial growth [5].

Many studies have been conducted to determine the effects of different dietary energy and protein levels on sheep growth performance. Mahgoub et al. [6] and Karim et al. [7] reported that average daily body weight gain and feed conversion efficiency were improved along with increasing dietary energy and protein levels. According to Ebrahimi et al. [5], feed intake was enhanced with increasing protein levels and declined with increasing energy levels. Besides, a number of feeding days have been reported to decrease with increasing protein levels and increase with increasing energy levels. As reported by Thomas et al. [8] on Merino lambs, increasing dietary protein levels would linearly increase dry matter intake and growth rate, which is supported by Dove and Milne [9]. This conforms to the findings reported by Titi et al*.* [10] and Haddad et al. [11], dietary protein with 16% crude protein (CP) is the optimum level for proper dry matter intake, daily weight gain, and feeding efficiency of Awassi sheep.

On the other hand, the reports of Gatenby and Sheep [12], as cited in Seid and Animut [13], reported that the mean minimum energy and protein levels at which the growing lambs do not lose weight are metabolizable energy (ME) of 9 MJ/kg dry matter (DM) and 8% CP or about 80 g/kg DM, respectively. In comparison, the growing lambs and lactating ewes need about 11% of CP or 110 g/kg DM for proper productive performance [12].

Ram lambs supplied to local markets from herders are 1–3 years of age with 10 kg mean hot carcass weight [14]. Thus far, most of on-station feeding trial research on ram lambs by supplementing concentrates on grass hay without determining their actual requirement. The feeds are formulated based on the accustomed proportions and concentrate supplementation levels in the academic and research stations. Such conventional feeding practices can result in overfeeding or underfeeding of animals, leading to uneconomical feedlot operation and affecting animals’ health. This indicates the requirements of better feed formulation and feeding systems to improve indigenous sheep’s growth performances. A few experiments have been conducted to evaluate the effects of different dietary energy and protein diets formulated in a complete feeding system under stall feeding of sheep and other ruminant animals in the country [2].

Hararghe highland and Afar sheep (AS) are among the prominent indigenous sheep breeds inhabiting high eastern land and low land agro-ecologies. These breeds’ preferences may have been due to the buyers’ adaptation to their body conformation, feed intake, and feeding efficiency [15]. Nevertheless, information on their optimum and maximum dietary energy and protein requirements for proper growth, nutrient use efficiency, and productivity in complete feeding systems is scanty. Furthermore, the studies so far conducted did not generate factual information concerning the manipulation of dietary energy and protein levels for both sheep breeds that would improve growth performance to achieve the desired slaughter live weights for the export market [15]. Therefore, this experiment was designed to evaluate the effects of feeding different levels of energy and protein diets on different growth performance parameters of Hararghe highland and AS. 

## Materials and Methods

### Ethical approval

All animal handling practices have followed the guidelines of treating animals, i.e., on Animal Ethics and Welfare in Behavioral research and teaching, despite an established system for animal experiments’ ethical approval in the university [16]. 

### Experimental site

The experiment was conducted at Haramaya University goat farm, which is located 515 km east of the capital, Addis Ababa. The site is situated at an altitude of 1,950 m above sea level, 9°25’N latitude, and 42°2’E longitude. It receives the mean annual rainfall of 790 mm and has the minimum and maximum temperatures of 9.73°C and 24.02°C, respectively [17].

### Animals and their management

Fifty animals, 25 from each breed and 12 months of age, and a mean IBW of 19.31 ± 1.7 kg were employed for this experiment. Hararghe highland sheep were purchased from *Deder* local market, whereas AS were purchased from the *Amibara* market. The age of the animals was determined by dentition and information from the owners. After reaching the research site, they were ear-tagged and quarantined for 21 days feeding on forage pasture during these periods. During this time, all lambs were de-wormed against internal parasites and sprayed acaricide to control external parasites. The lambs were vaccinated to protect the common diseases existing in the area, i.e., ovine pasteurellosis and anthrax, as per the recommendation for commercial sheep production [18]. 

### Preparation of experimental diets and feeding management

The experimental diets were formulated in a total mixed ration from WB, maize grain (MG), groundnut cake (GNC), pasture grass hay, table salt, ruminant vitamin, and mineral premixes (Table 1). The five dietary treatment groups having different combinations of dietary energy and protein levels [minimum Energy and Protein (mEmP), medium energy and protein (MEMP), medium Energy and high Protein (MEHP), high energy and medium protein (HEMP), and high Energy and high Protein (HEHP)] were formulated on an as-fed basis. The desired energy and protein levels in each diet were achieved by varying the quantity and quality of diet ingredients following the procedures of national research council (NRC) [19]. 

Ahead of commencing the actual experiment, the animals were assigned to each dietary treatment group and then acclimatized to diets’ and pens’ management for 2 weeks. The diets were offered in a separate trough being divided into two equal portions and provided at 08:00 AM and 04:00 PM on an as-fed basis. The diets were revised biweekly based on the attained body weight changes. Freshwater was freely offered to each lamb throughout the experimental period. Careful observation and recording for the occurrence of any health-related problems were carried out during the entire experimental period.

**Table 1.** Nutrient composition of experimental diets.

Variables	Energy and protein combinations in each treatment				
	mEmP2.388 Mcal10% CP	MEMP2.866 Mcal16% CP	MEHP2.866 Mcal20% CP	HEMP3.344 Mcal16% CP	HEHP3.344 Mcal20% CP
Physical composition (%)					
Grass Hay	50	40	34	16	19
Ground MG	18	26	21	48	40
WB	14	12	8	16	9
GNC	16	20	35	18	30
Table salt	1	1	1	1	1
Ruminant premixes	1	1	1	1	1
Chemical composition (%)					
Dry matter (DM)	93.85	92.46	91.35	90.44	90.15
OM	91.34	92.88	93.69	94.45	94.70
CP	10.07	15.64	19.64	15.72	19.77
NDF	55.05	48.70	42.10	47.36	47.86
ADF	19.11	16.49	13.38	15.03	14.28
ADL	6.70	4.34	3.37	3.02	2.80
Ash	8.66	7.12	6.31	5.55	5.30

ADF = acidic detergent fiber; ADL = acidic detergent lignin; NDF = neutral detergent fiber. Dietary treatments: (1) mEmP = minimum energy and protein; (2) MEMP = medium energy and protein; (3) MEHP = medium energy and high protein; (4) HEMP = high energy and medium protein; and (5) HEHP = high energy and protein.

### Research design and treatments

The animals from both sheep breeds were distributed randomly into five weight categories and five dietary treatments in a randomized complete block design with 2*5 factorial arrangements, in which five replications from each breed per block and treatment exist. The animals were placed in an individual pen set with feed and water troughs and cleaned every morning ahead of daily offering.

The two main effects were diets and breeds. The five dietary treatment groups were:

- Minimum energy and minimum protein diet (mEmP; 2.388 Mcal/kg DM and 10% CP). This diet was used as positive control, whereas the rest were test diets.
- Medium energy and Medium Protein diets (MEMP; 2.866 Mcal/kg DM and 16% CP)
- Medium energy and high protein (MEHP; 2.866 Mcal/kg DM and 20% CP)
- High energy and medium protein (HEMP; 3.344 Mcal/kg DM and 16% CP)
- HPHE; 3.344 Mcal/kg DM and 20% CP. Preparation of these dietary treatment groups was made following the recommended ranges of dietary energy and protein levels for sheep [20]. According to this author, diets having crude protein categories of < 120, 120–200, and >200 g/kg of DM are classified as low, medium, and high protein source diets, respectively. Besides, diets with metabolizable energy categories of <9, 9–12, and >12 megajoules per kilogram of dry matter are categorized as low, medium, and high-energy diets, respectively.

### Digestibility trial

A digestibility test was conducted using all animals in the respective treatments. Each lamb was fitted with fecal collection bags and acclimatized to carrying the bags for 3 days, and total feces collection was conducted for 7seven consecutive days. Feces collection was conducted in the morning, put in plastic sheet bags, then weighed and stored at −20°C. At the end of the collection period, fecal samples were pooled over each animal’s collection period, and 10% of the pooled samples were taken for the analysis. During these periods, daily feed offer and refusal were recorded for each animal. Then, dry matter intake was computed as the difference between feed offer and refusal. The apparent DM and nutrients digestibility coefficients (DCs) were calculated based on the method of McDonald et al. [21]: 

$$\mathrm{Apparent}\;\mathrm{DM}\;\mathrm{DC}=\frac{\mathrm{Fecal}\;\mathrm{DM}\;\mathrm{output}}{\mathrm{DMI}}\times 100$$

$$\mathrm{Apparent}\;\mathrm{nutrient}\;\mathrm{DC}=\frac{\mathrm{NI}-\mathrm{Fecal}\;\mathrm{NOP}}{\mathrm{Nutrient}\;\mathrm{int}\mathrm{ake}\;(\mathrm{NI})}\times 100$$

### Feed intake and body weight change

Daily diet offer and refusal were recorded for each animal throughout the experimental period. 

Then, the refusals were collected for each treatment every morning, and at the end of the experimental periods, composite samples per dietary treatment were taken for the analysis. Following this, DM and nutrient intake were calculated as the difference between amounts of feed offered and refusals on as fed basis. 

Each animal’s live weight was measured using a spring balance every 15 days after overnight diet withdrawal to account for differences in gut fill. Total body weight gain (TBWG) was computed as the difference between final and IBW. The average daily gain was calculated by dividing the TBWG by the number of feeding days.

Feed conversion efficiency (FCE) of experimental animals was determined by dividing average daily gains (ADG) by the amount of feed consumed per day. Besides, feed conversion ratio (FCR) was computed by dividing ADG by the amount of feed consumed per day. Metabolizable energy intake was determined by ME (Mcal /kg DM) = digestible energy (DE) × 0.82, while digestible energy was computed as DE (Mcal/kg DM) = total digestible nutrient intake (TDN) % × 0.04409 as described by NRC [19]. 

### Laboratory analysis

Chemical analysis of feed offer, refusal, and fecal samples collected during experimental periods was analyzed in Haramaya University, animal nutrition laboratory for their DM, Ash, organic matter (OM), and nitrogen (N) content as per the procedures of Association of official analytical chemists [22]. Similarly, the CP content was estimated as N * 6.25. Neutral detergent fiber (NDF), acid detergent fiber (ADF), and acid detergent lignin (ADL) were analyzed following the same procedures [22].

### Statistical analysis

The collected growth performance data were analyzed by using the general linear model procedures of statistical analysis software, version 9.4 [23]. The effects of breed and diets on the measured parameters were tested for significance by using the Tukey’s test to locate significantly different means. The statistical model used for the data set was: 

Yijkl = μ + Di + Bj + Bk + (D × B) ik + Eijkl. 

Where: 

Yijkl = the response variable; μ = overall mean; Di = effect of diets; Bj = effect of block; Bk = effect of breed; (D × B) ik = interaction between diets and breeds; Eijkl = the random error. 

Interaction and main effects were presented and discussed based on their existence. Bar graph plotting was conducted for body weight change across experimental periods using PROC GPLOT of SAS graph plotting.

## Results and Discussion

Proper understanding of animals’ dry matter intake and nutrients requirements is essential for the formulation of diets to prevent underfeeding or overfeeding of nutrients and promote efficient utilization of nutrients [30]. Nutrients feeding below the animals’ requirement would restrict growth, production, and affect their health. However, excess provision of nutrients results in more excretion of nutrients through feces and urine, and the left may be toxic and cause adverse health effects, even increases unnecessary feed costs [30]. As a result, a proper diet containing optimum dietary energy and protein levels promoting better growth performance and feeding efficiency had been identified in this experiment.

### Feed intake

The mean daily dry matter, organic matter, and crude protein intakes of ram lambs under all dietary treatment groups had shown significant (*p* < 0.01) difference in which MEHP *> *HEMP *> *HEHP *> *MEMP* >* mEmP. The mean daily DM intake as a percent of their body weight was higher (*p* < 0.01) for animals fed on MEHP diets, which is comparable with the recommended value of 2.6% [20]. However, the daily NDF, ADF, and ADL intake of experimental animals followed the opposite trend and was reduced as dietary energy and protein levels were increased. This implies that high fibrous nutrient (NDF) levels in the mEmP used as a positive control diet might affect the voluntary diet intake as a result of a slow rate rumen feed digestion and increased digesta retention time.

The research conducted on Iranian small-size Taleshi sheep fed on the diet containing metabolizable energy of 2.5 Mcal/kg DM and 14% CP recorded better results in daily dry matter intake (1,211.46 g) and dry matter intake as a percentage of their body weight (3.64%) as reported by Kiomarzi et al. [24]. This might be because local sheep with small body sizes are early maturing with high growth efficiency.

In this study, progressive diet intake was observed to increase the proportion of diet ingredients (GNC, WB, and MG) up to 60% of the test diet like in the MEHP diet (Table 2). In line with this study, NRC [19] and Asmare et al. [25] reported that animals feed intake increase when the concentrate levels in the diet increased up to 75% of diet composition, which minimizes the overall heat production in the rumen as compared with the high fibrous feed ingredients composition in the diet.

It seems that experimental diets formulated in total mixed ration had promoted dry matter and nutrient intakes due to its palatability, balanced nutrition, and its low rumen fill effect [26]. On the other hand, the mean daily DM, CP, and OM intake, and again their intake as a percentage of lambs body weight were higher (*p < *0.01) for Hararghe highland than AS (Table 2). Similarly, Hararghe highland sheep had higher (*p* < 0.01) mean daily dry matter intake expressed by metabolic body weight (44.6 g/BW^0.75^) than the AS (42.3 g/BW^0.75^). The current study results had indicated that the best dietary energy and protein levels for both sheep breeds would be MEMP (2.866 Mcal/kg DM with 16% CP). This was in agreement with the findings of Titi et al*.* [10] reported that 16% CP is the optimum dietary protein level for some sheep breeds. 

### Digestibility

The test diet containing MEHP diet improved the DC of DM, crude protein, and organic matter by 20.58%, 18.26%, and 12.12%, respectively. Similarly, higher (*p* < 0.01) digestibility of these nutrients was observed in animals fed on MEMP test diets. This might be due to the high soluble carbohydrate (starch and sugar) contents of these diets, which would result in more propionate production and a small amount of waste gas production (methane and carbon dioxide). However, a declining trend of these nutrients’ DC was observed on lambs subjected to HEMP and HEHP test diets. Besides, relatively the lowest digestibility of DM, OM, and CP, digestible energy, and %TDN intake was recorded under a positive control diet (mEmP). This might be due to its lower dietary energy and protein levels and higher fiber content than the other test diets. This affects microbial growth and feeds fermentation activities in the rumen [5]. Besides, there is an inverse correlation between the fiber content of diets (NDF) and its digestion rate. 

Similarly, lower digestibility of ADF and NDF was observed on the lambs subjected to higher dietary energy and protein levels (HEMP and HEHP) than MEMP and MEHP test diets. This might be due to a high proportion of grains concentrates (70%–80%) in HEMP and HEHP diets fed to lambs. Besides, it leads to lower rumen pH, which depresses cellulolysis and fiber digestion. In line with this, Ivey et al. [27] and Harikrishna et al. [28] reported that high caloric diets harm animal fiber digestion. In agreement with this study, higher inclusion of grain or concentrate ingredients to forage ingredient had attributed to acidic media in the stomach and cause digestive disorder, thereby reducing diet digestibility [21].

The most efficient digestible energy and %TDN intake in MEHP diets had shown balanced rumen degradable and bypass proteins and sufficient energy diet which enhanced rumen microbial growth and the fastest rate of substrates digestion (Table 3). This is concurrent with the findings of Mawati et al. [29], McDonald et al. [21], and McDonald et al. [30], stating that diet digestibility characteristics could be used as an essential parameter to determine its nutritional values and quality. Similarly, Kiomarzi et al. [24] and McDonald et al. [30] reported that diets with optimum energy and protein levels would promote high microbial populations, which attack the crude fiber more vigorously and facilitate rumen fermentation. On the other hand, nutrients (DM, OM, CP, NDF, and ADF) DCs were higher (*p* < 0.01) for Hararghe highland than AS. Similarly, Hararghe sheep had taken more digestible energy (DE Mcal/kg DM) of about 16.80% and TDN of approximately 16.81% than AS.

**Table 2.** Diet intake of Hararghe highland (*n* = 25) and Afar (*n* = 25) lambs.

Measurement	Breed		Diets						*p*-values		
	HHS	AS	mEmP	MEMP	MEHP	HEMP	HEHP	SEM	BR	DL	BR × DL
DMI (gm/day)	584.7^a^	503.0^b^	474.8^c^	510.7^bc^	620.6^a^	571.7^b^	542.5^bc^	4.09	**	**	ns
DMI (gm/BW^0.75^)	44.6^a^	42.3^b^	40.8^b^	40.6^b^	46.8^a^	44.4^ ab^	44.8^ ab^	0.15	**	**	ns
DMI (% BW)	2.9^a^	2.5^b^	2.4^c^	2.6^b^	2.9^a^	2.8^ab^	2.7^ab^	0.02	**	**	ns
CPI (gm/day)	119.8^a^	99.5^b^	92.4^b^	104.8^ab^	124.2^a^	114.7^ab^	110.1^ab^	1.00	**	**	ns
CPI (% BW)	0.37^a^	0.30^b^	0.28^b^	0.34^ab^	0.40^a^	0.37^ ab^	0.35^ ab^	0.003	**	**	ns
OMI (gm/day)	495.9^a^	444.8^b^	406.0^c^	443.5^b^	521.1^a^	486.9^ ab^	470.4^ab^	3.36	**	**	ns
OMI (% BW)	1.75^a^	1.42^b^	1.23^c^	1.40^bc^	1.76^a^	1.63^ ab^	1.50^b^	0.02	**	**	ns
NDFI (gm/day)	286.9^b^	338.5^a^	358.1^a^	329.2^a^	316.5^ab^	304.3^ab^	280.6^b^	2.98	*	*	ns
ADFI (gm/day)	108.9^b^	137.6^a^	152.6^a^	128.3^ ab^	118.2^ ab^	104.2^ab^	87.9^b^	1.17	*	*	ns
ADLI (gm/day)	40.0^b^	61.9^a^	65.3^a^	58.7^a^	54.3^a^	48.2^ab^	41.7^b^	1.12	*	*	ns

DMI = Dry matter intake; CPI = crude protein intake; OMI = organic matter intake; NDFI = neutral detergent fiber intake; ADFI = acid detergent fiber intake; ADLI = acid detergent lignin intake; HHS = hararghe highland sheep; AS = Afar sheep. Dietary treatments: (1) mEmP = minimum energy and protein (2.388 Mcal /kg DM and 10% CP); (2) MEMP = medium energy and protein (2.866 Mcal/kg DM and 16% CP); (3) MEHP = medium energy and high protein (2.866 Mcal/kg DM and 20% CP); (4) HEMP = high energy and medium protein diet (3.344 Mcal/kg DM and 16% CP); and (5) HEHP = high energy and protein (3.344 Mcal/kg DM and 20% CP). SEM = standard error of mean; BR = breed; DL = diet levels; ns = non-significant.

In each row, the numbers with different letters have a significant difference at *p* < 0.05 or *p* < 0.01.

### Live weight change

Breeds and all dietary treatments had shown significant (*p *< 0.01) differences in average daily gain, final body weight (FBW), and total gain except for the IBW (Table 4). Hararghe highland Sheep (HHS) was heavier (*p* < 0.01) by 4.3 and 3.1 kg in FBW and total gain, respectively, than AS. Similarly, the HHS was more efficient in nutrient utilization efficiency (FCE) (22.57 *vs.* 18.18), but less in FCR (4.20 *vs.* 5.50) as compared to AS. Moreover, HHS had shown higher (*p* < 0.05) performance in digestible and metabolizable energy and TDN utilization than AS. These variations might be due to their highland origin, which stimulated high dry matter intake and high digestibility rate with better nutrient utilization efficiency for live weight gain than Afar ram lambs [31].

Furthermore, AS having low dry matter intake and slow growth rate might be due to their low land area background. They require more energy to maintain their body temperature and lose heat quickly due to the greater surface area to volume ratio [32]. The energy lost by animals is either the energy in feces, urine, methane, or heat energy.

On the other hand, the highest (*p* < 0.01) FBW, total gain, gain rate, and ADG, and nutrients utilization efficiency (FCE) were recorded by ram lambs fed on the MEHP test diet followed by HEMP, HEHP, MEMP, and mEmP, respectively. The same results and trends were observed in metabolizable energy intakes (Mcal/kg DM) and TDN for all diet groups. This is possibly due to the adequate cereal grains and fibrous diet proportions included in MEHP diets to enhance lambs’ better performance in these regards. 

Various studies [5,10,32] have reported that medium caloric and high nitrogenous diets (MEHP) had resulted in higher body weight gain and growth rate of sheep. The findings of Kiomarzi et al. [24] in the experiment conducted on small Iranian Taleshi sheep indicated that the test diet with energy 2.5 Mcal ME kg^−1^ and 16% crude protein has resulted in better final weight (33.78 kg), average daily gain (145.69 gm) and feed efficiency (12.03). 

**Table 3.** Average digestible DM, nutrients intake, and digestibility coefficient of Hararghe (*n* = 25) and Afar (*n* = 25) lambs.

Measurements	Breed		Diet						*p*-values		
	HS	AS	mEmP	MEMP	MEHP	HEMP	HEHP	SEM	BR	DL	BR × DL
Digestible DM and nutrient intake (g)											
DMD	393.3^a^	334.4^b^	269.5^e^	327.3^d^	442.8^a^	403.2^b^	376.2^c^	3.27	**	**	ns
DCP	81.4^a^	66.3^b^	53.9^c^	65.6^b^	88.6^a^	78.8^b^	70.2^c^	1.05	**	**	ns
DOM	329.8^a^	284.9^b^	241.1^e^	286.2^d^	352.1^a^	331.0^b^	300.2^c^	2.32	**	**	ns
DNDF	179.3^b^	208.9^a^	220.6^a^	206.8^b^	197.8^ ab^	190.2^c^	174.3^d^	4.35	*	*	ns
DADF	71.4^b^	86.6^a^	102.6^a^	81.5^b^	72.7^c^	66.6^d^	56.8^e^	1.13	*	*	ns
Digestibility coefficient (%)											
DM	67.27^a^	66.48^b^	56.76^e^	64.09^d^	71.47^a^	70.52^b^	69.35^c^	0.35	**	**	ns
CP	67.98^a^	66.63^b^	58.31^e^	62.60^d^	71.34^a^	68.70^b^	63.76^c^	0.30	**	**	ns
OM	66.51^a^	64.05^b^	59.38^d^	64.53^b^	67.57^a^	67.10^ab^	63.82^c^	0.35	**	**	ns
NDF	62.51^a^	61.70^b^	61.60^ab^	62.82^a^	62.50^a^	62.51^a^	62.12^ab^	0.28	*	*	ns
ADF	65.52^a^	62.90^b^	67.23^a^	63.52^ab^	64.62^ab^	63.51^ab^	62.92^ab^	0.39	*	*	ns
Digestible energy (Mcal/kg DM)	2.44^a^	2.03^b^	1.67^e^	2.03^d^	2.64^a^	2.52^b^	2.36^c^	0.12	**	**	ns
TDN %	55.34^a^	46.04^b^	37.88^e^	46.04^d^	59.88^a^	57.16^b^	53.53^c^	2.45	**	**	ns

DMD = digestible dry matter; DCP = digestible crude protein; DOM = digestible organic matter; DNDF = digestible neutral detergent fiber; DADF = digestible acid detergent fiber intakes; DE = digestible energy; HHS = hararghe highland sheep; AS = Afar sheep. Dietary treatments: (1) mEmP = minimum energy and protein (2.388 Mcal/kg DM and 10% CP); (2) MEMP = medium energy and protein (2.866 Mcal/kg DM and 16% CP); (3) MEHP = medium energy and high protein diet (2.866 Mcal/kg DM and 20% CP); (4) HEMP = high energy and medium protein (3.344 Mcal/kg DM and 16 % CP); and (5) HEHP = high energy and protein (3.344 Mcal/kg DM and 20% CP). SEM = standard error of mean; BR = breed; DL = diet levels; ns = non-significant.

In each row, the numbers with different letters have a significant difference at *p* < 0.05 or *p* < 0.01.

**Table 4.** Nutrient utilization and body weight change of Hararghe (*n* = 25) and Afar (*n* = 25) lambs.

Measurements	Breed		Diet					*p*-values			
	HS	AS	mEmP	MEMP	MEHP	HEMP	HEHP	SEM	BR	DL	BR × DL
IBW (kg)	19.9	18.67	18.8	18.9	18.9	18.7	18.6	0.18	ns	ns	ns
FBW, kg	31.2^a^	26.9^b^	26.4^e^	29.3^c^	31.3^a^	30.2^b^	27.8^d^	0.20	**	**	ns
Metabolic body weight (BW^0.75^)	13.20^a^	11.81^b^	11.65^b^	12.59^ab^	13.23^a^	12.88^ab^	12.11^b^	0.12	**	**	ns
TBWG, kg	11.3^a^	8.23^b^	7.6^e^	10.4^c^	12.9^a^	11.7^b^	9.2^d^	0.10	**	**	ns
Gain rate (TBWG/FBW)	36.2^a^	30.6^b^	28.8^e^	35.5^c^	41.2^a^	38.7^b^	33.1^d^	0.34	**	**	ns
Average daily gain (gADG)	124.4^a^	91.4^b^	84.4^c^	115.6^bc^	143.3^a^	130.0^ab^	102.2^b^	1.05	**	**	ns
FCE (gADG/gDMI)x100	22.57^a^	18.18^b^	17.77^c^	22.64^ab^	23.13^a^	22.74^ab^	18.84^b^	0.25	**	**	ns
FCR (gDMI/gADG)	4.20^b^	5.50^a^	5.63^a^	4.42^ab^	4.32^b^	4.40^ab^	5.31^c^	0.07	**	**	ns
Metabolizable Energy (Mcal/kg DM)	2.01^a^	1.68^b^	1.37^e^	1.66^d^	2.17^a^	2.07^b^	1.94^c^	0.35	**	**	ns

FCE = feed conversion efficiency; FCR = feed conversion ratio; HHS = Hararghe highland sheep; AS = Afar sheep. Dietary treatments: (1) mEmP = minimum energy and protein (2.388 Mcal/kg DM and10% CP); (2) MEMP = medium energy and protein (2.866 Mcal/kg DM and 16% CP); (3) MEHP = medium energy and high protein (2.866 Mcal/kg DM and 20% CP); (4) HEMP = high energy and medium protein (3.344 Mcal/kg DM and 16 %CP); and (5) HEHP = high energy and protein diet (3.344 Mcal/kg DM and 20% CP). SEM = standard error of mean; BR = breed; DL = diet levels; ns = non-significant.

In each row, the numbers with different letters have a significant difference at *p* < 0.05 or *p* < 0.01.

Moreover, high caloric and high nitrogenous (HEHP) diets composed of high grain and lower fibrous diet ingredients would reduce of converting the rate of DE–ME as it increases urine energy through more urea production [5]. Similarly, this study showed less efficient utilization of nutrients for a given daily live weight gain in sheep with a higher level of dietary energy and protein (HEMP and HEHP) than the other treatments. In agreement with this, Ahmed [33] and Ebrahimi et al*.* [5] reported that feeding efficiency was inversely related to the diets’ energy and protein levels. According to these authors, the low feeding efficiency with high protein levels in the diets might be due to the surplus amino acids, which need several processing reactions to be converted into other useful compounds. Each reaction produces heat energy. Hence, animal body weight gain and products are negatively affected due to the reduced rate of microbial protein synthesis and total protein supply to the animals.

In this study, ram lambs fed on the MEMP diet had relatively better body weight gain than those fed on HEMP and HEHP diets in many aspects. Accordingly, it is the optimum test diet for promoting proper growth and nutrient utilization without incurring feeding costs in both breeds of lambs. This is likely why a fast growth rate was observed when increasing the energy and protein levels from mEmP to MEMP. Besides, lambs’ proper energy and protein requirements were met at optimum dietary energy and protein levels [5]. However, slow growth was observed when energy and protein levels increased from MEHP to HEMP and HEHP diets. The optimum level of dietary protein for tropical sheep breeds is 16%, as reported by Titi et al. [10] and Haddad et al. [11]. Moreover, in the diet with metabolizable energy < 2.50 Mcal/kg DM, the extra caloric effect of increased protein levels (16% CP) in the diet has resulted in higher average daily gain and better lambs’ performance. It is reported that when the protein supply exceeds the requirement, energy becomes a limiting factor for growth, and the animals no longer respond to additional intakes of protein [34].

**Figure 1.**
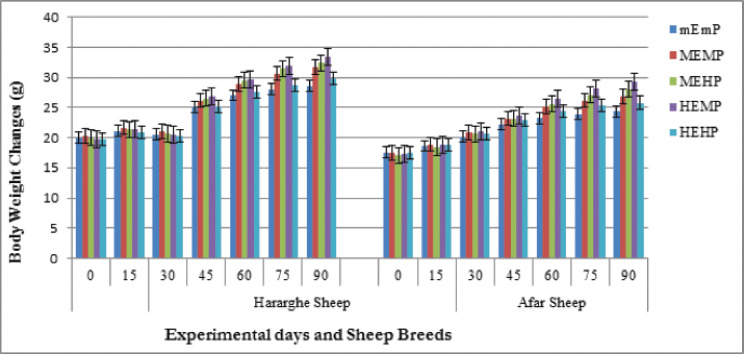
Biweekly live weight change of sheep breeds subjected to different diets.

Figure 1 shows the live weight changes measured every 15 days of the experimental period. The measured live weight change in under all treatments was similar up to the first 15 days of feeding trials, and the diet effect was observed after these days. It is clear from Figure 1 that body weight gain profile across feeding times for the animals fed on all dietary treatments was slightly increased at the first and second 15 days of the experimental period. After that, the MEHP and HEMP dies weight gain continued to increase progressively until the end of the feeding trial. But MEMP and HEHP dietary treatments showed a slight linear increment, whereas the weight gain in the control treatment (mEmP) increased slightly. The figure indicated that steady state and accelerated body weight changes per 15 days were observed on Hararghe sheep until 30 days and after 45 days of feeding trial, whereas AS had shown slow and linear body weight gain across the feeding duration.

On the other hand, Ayele and Urge [31] had reviewed most of the stall feeding works of literature on indigenous Ethiopian sheep breeds. They indicated that sheep breeds fed on various types and levels of basal and supplement diets recorded an average daily gain of 71 gm. Moreover, these sheep managed on such a feeding system not attained the slaughter market weight of 25 kg at finishing ages of 1 year and 4 months as required by the export market. 

Hence, compared to the previous studies conducted on stall feeding of sheep breeds in the country, the current results indicated that experimental animals fed on all test diets (MEMP, MEHP, HEMP, and HEHP), including the control diet (mEmP), have performed well. This might be due to diets formulated in a total mixed ration, which has promoted dry matter and nutrient intakes, feeding efficiency, and fast growth due to better palatability, balanced nutrition, and the low rumen fill effect. In this study, the adequate metabolizable and digestible energy (Mcal/kg DM) intake and total digestible nutrients for all lambs under all dietary treatment groups implied better energy efficiency in the animals’ bodies.

## Conclusion

Hararghe highland sheep had better performance in dry matter and nutrients intakes, live weight gain, and feeding efficiency than AS. Besides, significant effects of diets observed with the ram lambs fed on MEHP (2.866 Mcal ME kg^−1^ and 20% CP) diet had shown maximum growth performances. On the contrary, the declining trend was observed on the lambs subjected to HEMP and HEHP diets despite incurring costs of feeding and management. However, lambs fed on MEMP (2.866 Mcal ME kg^−1^ and 16% CP) had shown medium growth performances with the optimum cost of feeding and management compared to MEHP, HEMP, and HEHP diets. Hence, it is optimum diets for desirable growth performance and normal physiological needs (without the digestive disorder) for both sheep breeds. Thus, entrepreneurs keeping sheep for large-scale fattening are advised to feed diets with sufficient energy and protein levels, resulting in higher feed conversion efficiency without incurring feeding and veterinary services costs.

## List of Abbreviations

ADF: Acid detergent fiber; ADG: Average daily gain, ADL: Acid detergent lignin, CP: Crude protein, DC: Digestibility coefficient, FBW: Final body weight, FCE: Feed conversion efficiency, FCR: Feed conversion ratio, g: Grams, GNC: Groundnut cake, HHS: Haraghe highland sheep, IBW: Initial body weight, ME: Metabolizable energy, MG: Maize grain, N: Nitrogen, NDF: Neutral detergent fiber, NRC: National Research Council, OM: Organic matter, TBWG: Total body weight gain, TDN: Total digestible nutrient, WB: Wheat bran.
